# Development of a chronic compression spinal cord injury model in neonatal and adult rats

**DOI:** 10.1002/ame2.12484

**Published:** 2024-09-18

**Authors:** Reggie Ridlen, Victoria Marsters, Elizabeth Clarke, Kristine McGrath, Catherine A. Gorrie

**Affiliations:** ^1^ School of Life Sciences University of Technology Sydney Sydney Australia; ^2^ Kolling Institute of Medical Research, Sydney Medical School University of Sydney Sydney Australia

**Keywords:** animal models, neonatal, neuroscience, reproducibility, spinal cord

## Abstract

**Background:**

Spinal cord injury presents a significant burden globally, with traumatic spinal cord injury being the predominant cause historically. However, nontraumatic spinal cord injury (NTSCI) is emerging as a significant contributor, particularly in developed nations, yet it remains poorly understood due to underreporting and misclassification. NTSCI, spanning various etiologies such as bony growths, vascular conditions, infections, neoplastic conditions, and immune disorders, poses unique challenges in diagnosis and treatment, often resulting in lifelong morbidity. This study addresses the lack of suitable animal models for NTSCI research, especially in neonatal animals.

**Methods:**

Utilizing a solid spacer approach, we developed a compression NTSCI model applicable to both neonatal and adult Sprague–Dawley rats.

**Results:**

Through anatomical measurements and in vivo experiments, we confirmed the feasibility and safety of the spacer insertion procedure and observed no acute off‐target effects.

**Conclusion:**

The versatility of this model lies in its adaptability to different ages of rats, offering a cost‐effective and reproducible means to induce graded injuries. Moreover, behavioral assessments demonstrated observable hindlimb function, validating the model's utility for studying functional outcomes. Although challenges persist, particularly in accounting for spinal column growth in neonatal animals, this model fills a crucial gap in pediatric NTSCI research. By providing a platform to investigate pathophysiological mechanisms and test potential treatments, it offers promising avenues for advancing our understanding and management of pediatric NTSCI.

## Graphical abstract

Image 1: T11 laminectomy T10; alignment over spinal cord (SC); placement on SC; angled under T10 and inserted; placed under T10; T10 spinal cord compression hindlimb motor impairment. Image 2: (1) Anatomical measurements: three‐dimensional printed spacer inserts that can be adjusted to occlude 50% of the spinal cord at any age. Image 3: (2) Surgical implantation: T11 laminectomy at postnatal days P7, P9, and P14, and 9 weeks to insert the spacer. Image 4: (3) Behavioral assessments: measurable behavioral outcomes due to spinal cord compression.

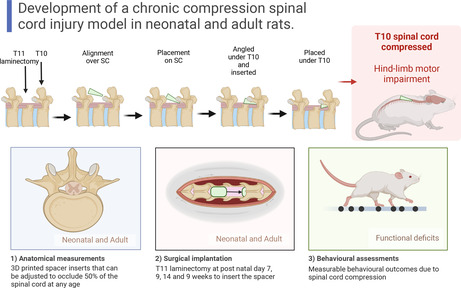

## 1 INTRODUCTION

Spinal cord injury (SCI) is a devastating condition that can cause severe morbidity across all ages and socioeconomic groups. The reported global SCI incidence was 0.9 million new cases per year in 2019, with a total prevalence of 20.6 million.^1^ The most common cause of SCI is traumatic spinal cord injury (TSCI), which had an incidence of 12–60 cases per million people in 2019, with an increasing worldwide incidence over the past 30 years.^1^, ^2^ However, it is believed that SCIs from nontraumatic causes (NTSCI [nontraumatic spinal cord injury]) have an increasing incidence, especially in developed nations, and have been reported to have a mortality rate of 1.62 times greater than the general population.^3^, ^4^, ^5^ The exact global incidence of NTSCI is unknown due to poorer reporting and classification. However, recent studies have indicated that the prevalence of NTSCI from all causes may be higher than previously thought and closing the gap with TSCI,^6^, ^7^, ^8^ and NTSCI resulting from various causes, such as bony growths, vascular conditions, infections, neoplastic conditions, and immune disorders, have gone largely under‐researched.^3^, ^4^ The age demographics for NTSCI are also quite different from those for TSCI, with a large incidence occurring in ages ≥55 years and a higher percentage of overall cause of SCI (66%) in neonatal/young children (≤12 years old) compared to 20% for the adult population.^9^, ^10^

NTSCI is hard to classify at the clinical level, as the disease is often treated only by the cause, not the injury type.^11^, ^12^ Conditions such as tumors or immune disorders affecting the spinal cord often get treated only for their onset, with little guidance or intervention to recover the affected SCI post‐removal of the cause.^7^ This leaves lifelong morbidity due to the underlying damage caused by NTSCI, only worsening over time due to tissue necrosis and poor circulation if improperly treated.^13^ This chronic underreporting and misclassification of NTSCI across all age demographics further the need for new clinical treatments and interventions beyond removing the initial injury.^7^ There are many subclassifications and types of NTSCI,^14^ the most common of which is compression injuries. Compression NTSCI, such as spondylitis or tumors, chronically compresses the spinal cord over time and requires immediate decompression.^13^ These conditions are an example of primary treatment, with no secondary intervention to reverse or treat the damage already caused.

The etiology and epidemiology of this type of SCI are not well understood and have been under‐researched.^9^ This has affected the modeling of NTSCI in animals, with no current replicable, accurate, or reliable models for adult and neonatal animals.^6^, ^9^ Therefore, it is pertinent to develop a model of NTSCI useful for neonatal and adult research so that we can study the pathophysiology and develop and test potential treatments/interventions for these types of SCIs.

After all available preexisting models of NTSCI in the literature were reviewed,^15^ we selected a solid spacer approach to develop a compression SCI in neonatal and adult rodents. There are reports of spacer compression being used in adult rats and mice under different experimental conditions and with different designs and materials.^16^, ^17^, ^18^, ^19^ Advances in three‐dimensional (3D) printing have improved the ease, reproducibility, and adaptability of spacer production, allowing the development of spacers suitable for use in neonatal and adult rats, and the range of available materials includes biological inert substances such as polylactic acid (PLA). We believe that this method of compression NTSCI will provide a reproducible and less‐variable model of short‐term and prolonged thoracic NTSCI in neonatal and adult Sprague–Dawley (SD) rats. This study describes a proof‐of‐concept investigation and safety study to ensure that this model is feasible. It is therefore conducted only at early time points..

## 2 METHODS

### 2.1 Animal welfare

A total of 84 neonatal and adult mixed‐sex SD rats were born in‐house from time‐mated dams obtained from the Animal Resource Centre (ARC) in Perth, Western Australia, and housed in the Ernst Facility (University of Technology Sydney [UTS]). These experiments were approved by the UTS Animal Care and Ethics Committee (ETH21‐6694). Animals were provided with standard rat chow and housed under a 24‐h dark–light cycle with environmental enrichment. Litters of 12–16 pups were housed together with their mother and removed in small groups of litter mates at the appropriate time points for experimental surgery at P7, P9, and P14, and 9 weeks (adult) or for behavioral tests. Sample sizes are deliberately small with a minimum sample size of *n* = 3–4 per group to address the 3Rs (replacement, reduction, and refinement) of animal ethics. Animals were randomly assigned an identifying number at birth for identification throughout the experiment. These rats were part of two experimental projects: (1) spinal column size analysis and (2) 1‐, 3‐, and 7‐day safety and survival compression studies.

#### 2.1.1 Development of the solid spacer

**FIGURE 1:**
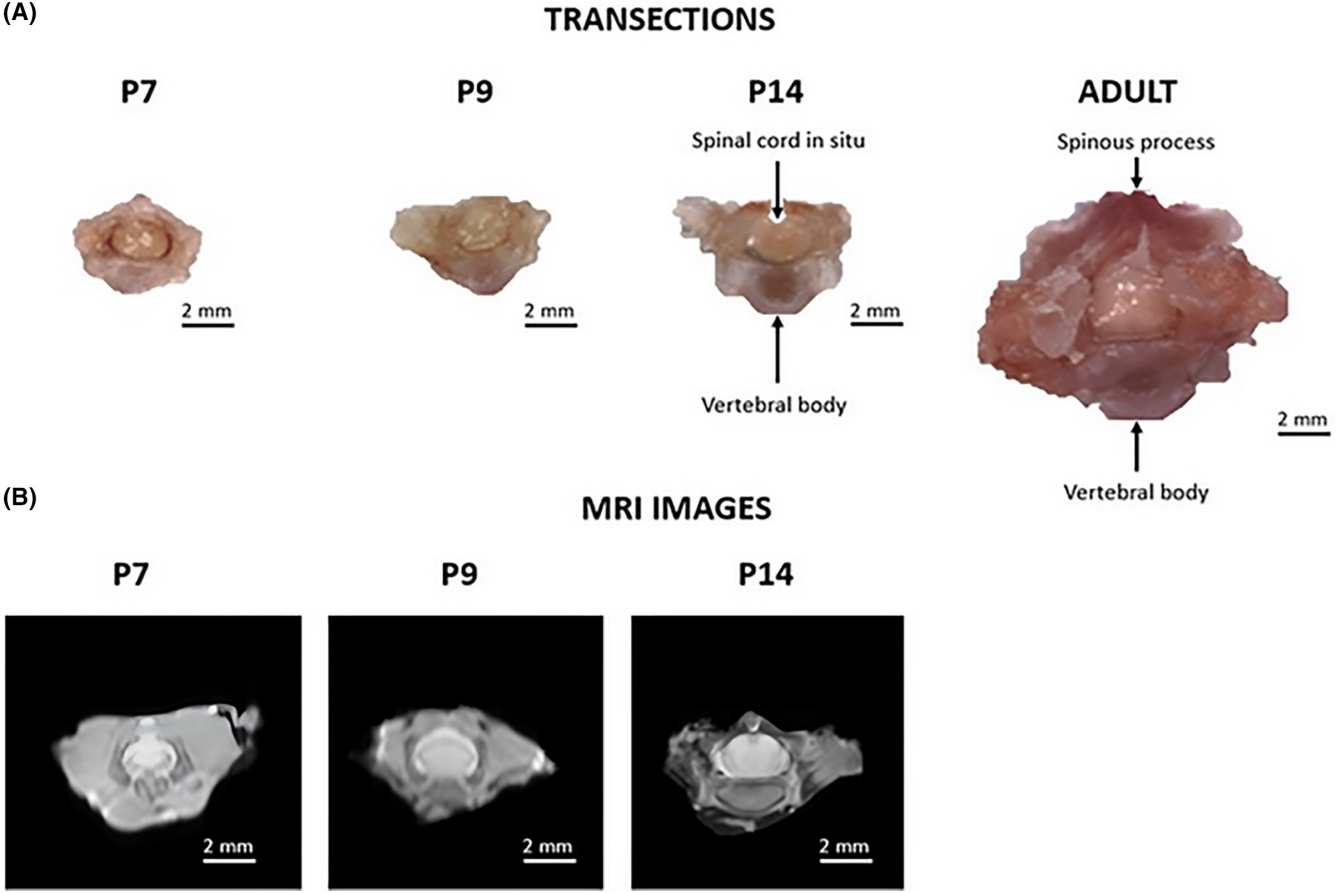
(A) Cross sections of perfused surgically removed thoracic vertebra 10 for postnatal day P7, P9, and P14, and 9‐week (adult) Sprague–Dawley (SD) rats and (B) cross sections of ex vivo spinal columns with the spinal cord in situ imaged using MRI (magnetic resonance imaging) for postnatal days P7, P9, and P14 SD rats.

Normal SD rats (*n* = 24) were used to measure the parameters of the spinal column with spinal cord in situ. Groups of three male and three female rats were used at postnatal days P7, P9, and P14, and 9 weeks (adult). Animals were euthanized using lethal injection (pentobarbitone, 1 mL/kg, i.p. [intraperitoneal]) and then subjected to cardiac perfusion with heparinized saline and 4% paraformaldehyde (PFA).^20^ Spinal columns between T8 and 12 were removed and the T10 vertebra was carefully dissected with the spinal cord in situ (Figure 1A) and used to measure the height, width, and area of the spinal column and spinal cord. Ex vivo magnetic resonance imaging (MRI, Bruker BioSpec Avance III 94/20) was used in a subset of three vertebral columns (Figure 1B) to confirm that there was no shrinkage caused by the fixation process using PFA at ages P7, P9, and P14.^21^

Using these measurements, solid spacers were developed for neonatal and adult rats based on the design by Dimar et al.^17^ and Batchelor,^16^ who reported that a 50% spinal cord compression was needed to elicit a measurable deficit in adult SD rats at thoracic vertebra 10 (T10).^16^, ^17^ 3D spacers were subsequently printed with PLA using a Cacoon Create printer (Supp 1).

#### 2.1.2 Short‐term safety and survival studies

Short‐term safety and survival studies were conducted to ensure no unwanted effects were associated with the surgery or spacer insertion. Compression surgery was undertaken in mixed‐sex neonatal rats (*n* = 3–4) at P7, P9, and P14, and 9 weeks (adult) (3F:3M) with postsurgical recovery for 1, 3, or 7 days. At each stage, animals were monitored carefully for hindlimb and forelimb deficits, infection, and inflammation at the wound site. We further assessed each animal postmortem to study the position of the spacer and any other unexpected or adverse effects from surgery on the surrounding tissue and spinal cord. This study aimed to understand recovery patterns and injury severity, measure the motor function impact over several short time points, and ensure there was no excessive hemorrhage or deleterious events that would be the cause of or result from an acute type of injury.

### 2.2 Surgical procedures

Experimental animals underwent a surgically induced NTSCI using the 50% age‐appropriate spacer. Animals were anesthetized with 5% isoflurane supplemented with 1 L/min oxygen for 5 min prior to surgery. Anesthesia was maintained between 2% and 4% throughout surgery, depending on the animal's vital signs. Prior to the procedure, animals were administered analgesics (buprenorphine hydrochloride Temgesic, infants: 0.01 mg/kg, adults: 0.03 mg/kg), antibiotics (cefazolin sodium: 33 mg/Kg), local anesthetic (bupivacaine: ⁓0.1 mL), and Hartman's fluid replacement solution (compound sodium lactate: 15 mL/kg) via subcutaneous injection.

**FIGURE 2:**
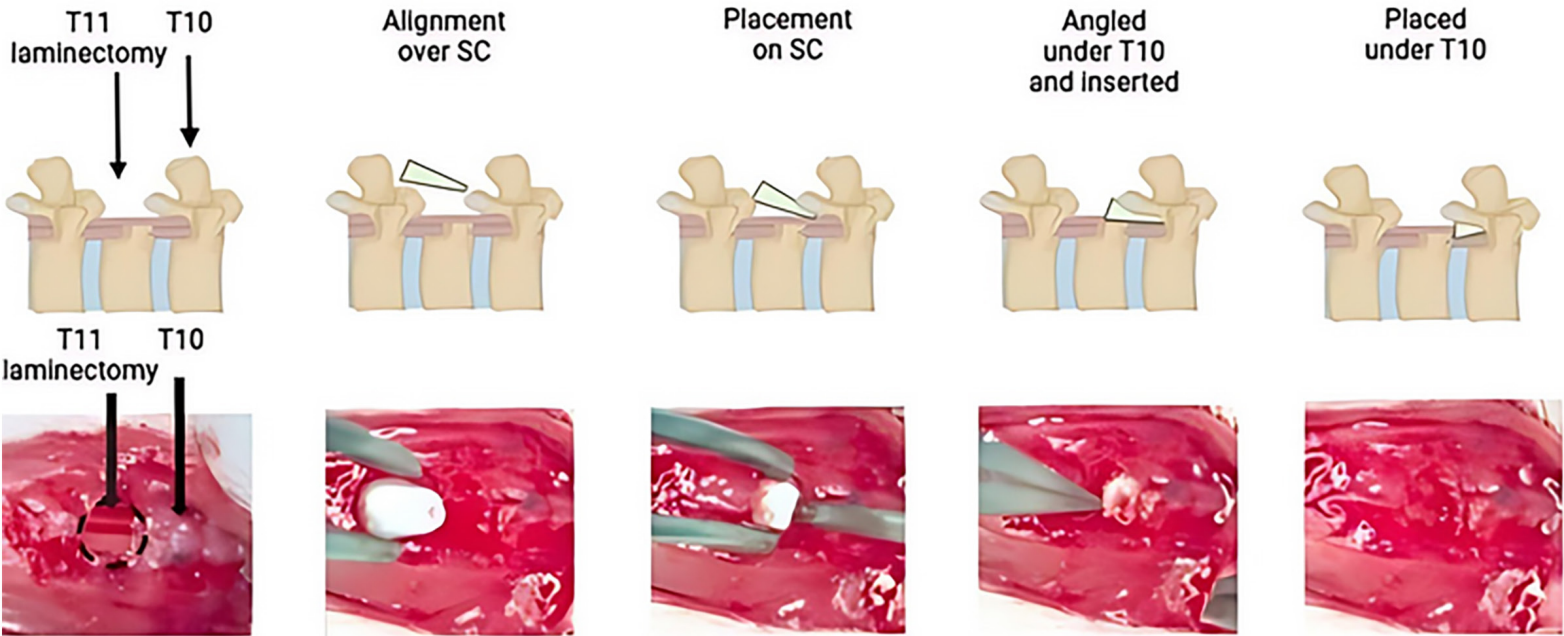
Insertion of a wedged‐shaped polylactic acid spacer under thoracic vertebra 10 after laminectomy of thoracic vertebra 11. The spacer is inserted between the dorsal spinal column and the dura of the spinal cord (SC). The spacer can be left in for days to weeks. Created with BioRender.com.

A surgical incision was made through the skin at the dorsal midline, from the mid‐ to the lower thoracic region. Subsequent tissue layers were then carefully opened in layers using fine curve–tipped forceps and fine shape scissors until the spinal column was exposed and visible. T10/T11 was identified by its shape and location, followed by a bilateral laminectomy to expose the dura of the spinal cord. The compression injury using the 3D spacer was placed within the opening formed in the vertebral column. The spacer was placed resting on the surface of the dura with the ventral side down and pointing toward T10. The spacer was then gently moved under T10 using fine curve–tipped forceps so that the spacer was barely visible (Figure 2).

After injury, fascia, muscles, and skin were closed using 4–0 Monocryl Sutures and VetBond glue. Infant rats were cleaned thoroughly with saline and exposed to littermates and bedding material before being returned to their mothers for postoperative care.^22^ Adult rats were placed back in their home cage and given subcutaneous analgesics and assisted bladder expression every 12 h for 3 days (if applicable).

All experimental animals were euthanized using lethal injection (pentobarbitone, 1 mL/kg, i.p.) and subjected to cardiac perfusion with heparinized saline and 4% PFA.^20^ The spinal cord between T8 and L1 was removed, placed in individual centrifuge tubes stored in 4% PFA for 24 h, and then transferred to a 30% sucrose solution with 0.02% sodium azide.

### 2.3 Locomotor function

Motor function was assessed at 1, 3, and 7 days postinjury using different methods. For neonatal rats, motor function was assessed in an open field using a six‐point overground locomotor score for infant rats.^23^ Motor function was also assessed in neonates using an air‐stepping (AS) apparatus described by Yang and Jamon to measure the movement and coordination of the hind limbs.^24^, ^25^ This removed the possibility of movement associated with neuronal excitement in the hind limbs due to external sensory stimulation.^26^, ^27^, ^28^ The AS test (Table 1) was quantified using a semiquantitative scoring method, which measured the range of movement of each joint of the hind legs, as well as coordination between the joints and hind legs.

**TABLE 1:** Scoring methodology for neonatal air‐stepping behavioral assessment.

Test	Criteria	Score (total left + right)
Involvement in movement: scoring each joint of the hind limbs separately (hip, knee, ankle) for each hind limb (left and right)	No movement (0), slight or occasional movement (0.5), often or extensive movement (1)	/6
Involvement in cycling: scoring each joint of the hind limbs separately (hip, knee, ankle) for each hind limb (left and right)	No cycling (0), slight or occasional cycling (1.5), sustained cycling (3)	/6
Involvement in grasping: scoring each hind limb separately (left and right)	No movement (0), slight or occasional movement (0.5), often or extensive movement (1)	/2
Front limb/hind limb synchronization: scoring each hind limb separately (left and right)	No synchronization (0), slight or occasional synchronizing (0.5), often or extensive synchronized movement (1)	/2
Hind limb coordination: scoring each hind limb separately (between left and right)	No movement (0), slight or occasional flailing movement (0.5), often or extensive coordinated movement (1)	/1
Total score		/17

For adult rats, hindlimb motor function was assessed in an open field using the 21‐point BBB (Basso, Beattie, and Bresnahan) hindlimb scoring method.^29^ Motor function was also assessed in adult rats using a horizontal ladder task that was used to measure the percentage of correct hindlimb foot placements over irregularly placed rungs.^30^

### 2.4 Sectioning and histology

Spinal cord tissue (1 cm) centered over the lesion site was sectioned (16 μm thick) transversely rostral to caudal using a Thermo NX70 cryostat, collecting every fifth section. Fourteen sections were mounted on a series of 10 slides, providing a cross section of the injury through the lesion site.

The first slide in each series was rehydrated for 10 min in PBST (phosphate‐buffered saline Tween‐20) buffer before using the standard protocol for hematoxylin and eosin (H&E) to stain the tissue. Images of slides were obtained using a Zeiss Axioscan Slide‐Scanning microscope, high throughput at 20× magnification, with the black balance adjusted to control for variance between stains. H&E stains were used to identify the epicenter of injury on each slide and were graded on several criteria (Table 2) to develop a global injury score.

**TABLE 2:** Scoring methodology for T10 spinal cord H&E‐stained sections.

Test	Criteria	Score
Dorsal compression	No compression (0), light indentation (1), obvious indentation (2), severe compression (3)	/3
Axonal swelling	No swollen axons (0), occasional (<10) swollen axons present in small amounts (1), several (11–50) swollen axons, numerous (>50) swollen axons are seen (3)	/3
Gray matter disruption	No disruption present (0), cell disruption less than one fourth of area (1), cell disruption over one fourth of area but less than one half (2), severe cavitation, over one half of area (3)	/3
White matter disruption	No disruption present (0), cell disruption less than one fourth of area (1), cell disruption over one fourth of area but less than one half (2), severe cavitation, over one half of area (3)	/3
Central canal disruption	No disruption present (0), central canal is damaged but still distinguishable (1), central canal is no longer circular, but the cells are identifiable or are moderately swollen (2), central canal cannot be identified or seen, or is considerably swollen (3)	/3
Shift/tissue deformation	No tissue shift (0), some shifting/deformation (1), large tissue shifts or deformation (2), whole tissue is shifted and deformed (3)	/3
Hemorrhage	No hemorrhage (0), occasional red blood cells visible in tissue (1), mild hemorrhage (2), obvious and substantial hemorrhage visible (3)	/3

*Note*: Scoring method: 0, normal spinal cord tissue; 1, a slight change in morphology; 2, an obvious deformation of the tissue; and 3, severe deformation of the spinal cord tissue.

Abbreviation: H&E, hematoxylin and eosin.

## 3 RESULTS

### 3.1 Spacer design

#### 3.1.1 Sex difference

**FIGURE 3:**
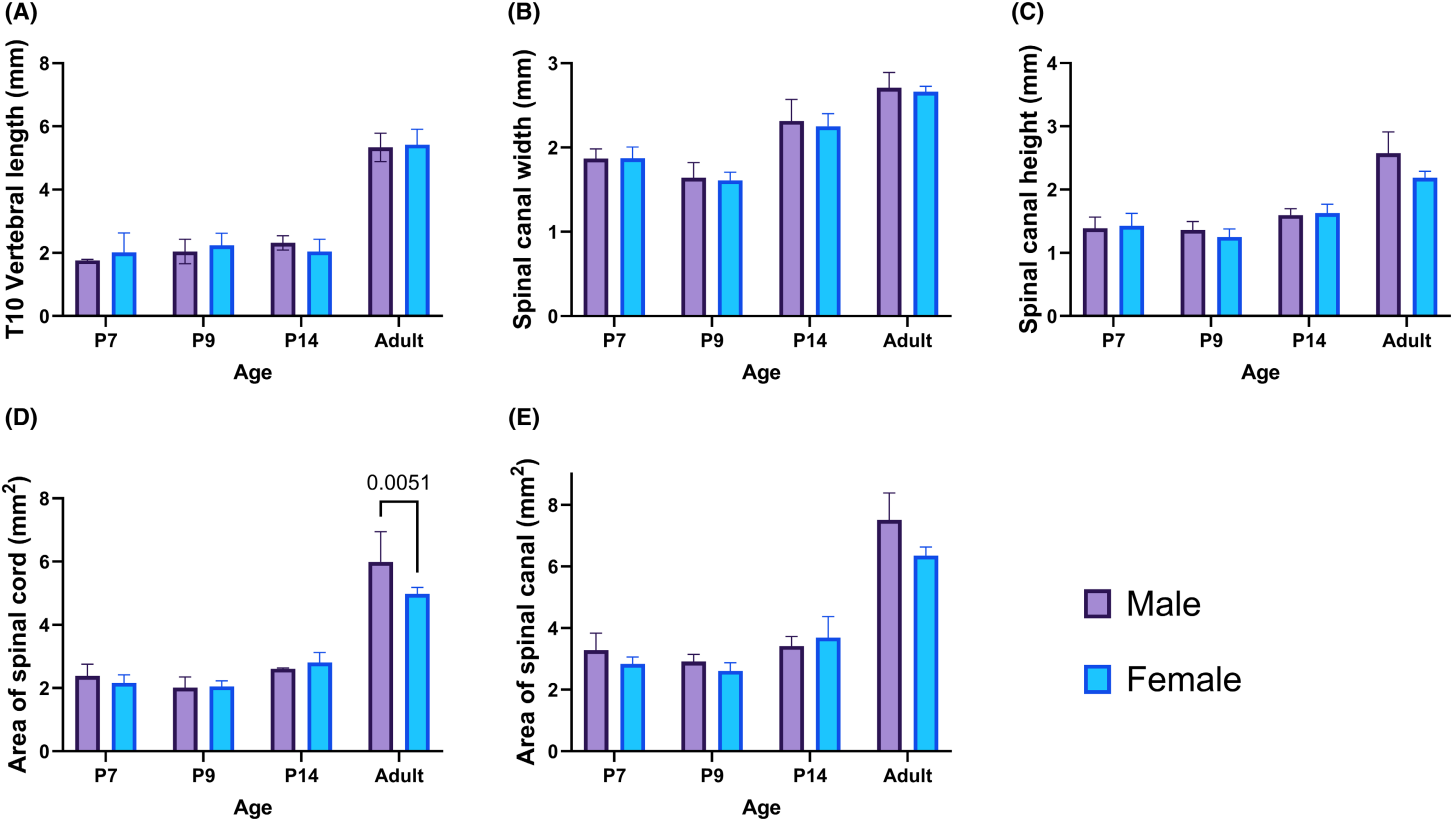
Sex differences (*n* = 3 M:3F) of thoracic vertebra 10: (A) vertebral length, (B) spinal canal width, (C) spinal canal height, (D) total spinal cord area, and (E) spinal canal area at postnatal day P7, P9, and P14, and 9‐weeks (adult) Sprague–Dawley rats. Analysis was conducted using two‐way ANOVA (analysis of variance) and is shown with mean ± standard deviation. Significant differences below *p* < 0.05 using Bonferroni post hoc tests are shown numerically.

There were no significant sex differences in T10 vertebral length, spinal canal width, or height for any of the age groups (Figure 3A–C). There were no significant sex differences in spinal column area or spinal cord area for the neonates (Figure 3D,E). Male adult rats had significantly higher spinal column area and spinal cord area compared to the female rats (analysis of variance [ANOVA], Bonferroni post hoc tests, *p* < 0.5) (Figure 3D,E).

As expected, all measurements were significantly higher in the adult rats compared to the neonatal rats (*p* < 0.0001). Measurements for vertebral width and height were significantly lower in the P9 group compared to the P7 and/or P14 neonatal groups (*p* < 0.05), but there was no difference in spinal column area or spinal cord area between the three neonatal age groups. The percentage of spinal cord within the spinal column was consistent at about 76% for all groups (Table 3).

**TABLE 3:** Average T10 vertebra measurements for four age groups.

	P7	P9	P14	Adult
Length of T10 (mm)	1.95 ± 0.48	2.08 ± 0.31	2.20 ± 0.29	5.38 ± 0.38*
Width of spinal canal (mm)	1.86 ± 0.12	1.62 ± 0.12**	2.28 ± 0.18	2.69 ± 0.11*
Height of spinal canal (mm)	1.40 ± 0.18	1.30 ± 0.12***	1.66 ± 0.11	2.38 ± 0.28*
Area of spinal cord (mm^2^)	2.20 ± 0.27	2.03 ± 0.22	2.71 ± 0.21	5.48 ± 0.76*
Area of spinal canal (mm^2^)	2.92 ± 0.35	2.74 ± 0.26	3.55 ± 0.45	6.93 ± 0.78*
% Spinal cord within spinal canal	79 ± 0.05	76 ± 0.05	74 ± 0.08	75 ± 0.05

* *p* < 0.0001 compared to each of the neonatal rat age groups.

** *p* < 0.05 compared to P9 and P14.

*** *p* < 0.05 compared to P14.

MRI was used to determine if postmortem perfusing and tissue fixing affected the size of the spinal cord. Due to the small size of the neonatal spinal cords, MRI resolution was low, which made it difficult to measure the spinal columns and cords (Figure 1B). However, we found that there were no significant changes in any of the previously used dimensions using fixed tissue (*n* = 3 females) and fresh tissue (*n* = 3 females) at P7, P9, and P14.

#### 3.1.2 Spacer dimensions

**FIGURE 4:**
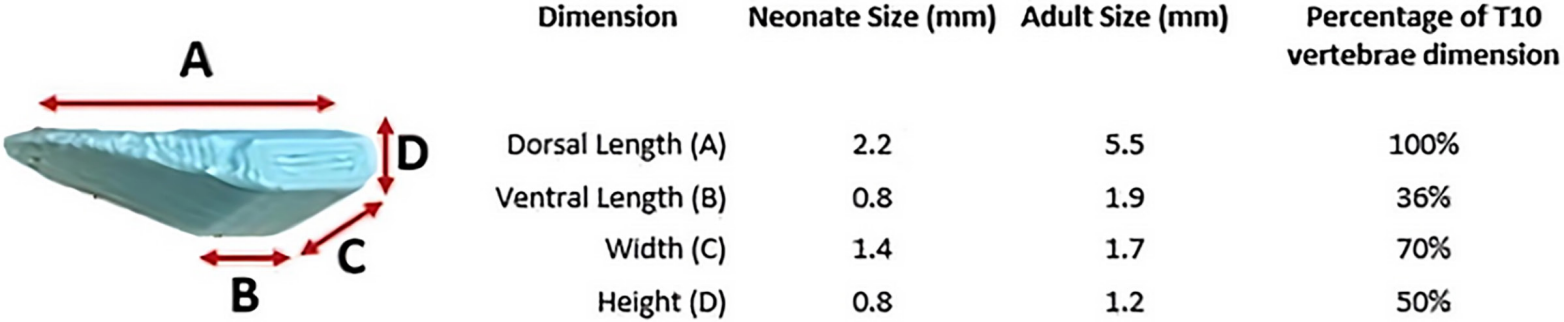
3D (three‐dimensional) printed PLA (polylactic acid) spacer dimensions for insertion under the T10 vertebra for neonatal and adult Sprague–Dawley (SD) rats. Lengths (A–D) and percentage occlusions for a neonatal (postnatal days P7, P9, and P14) and adult SD rats are shown as total length (in mm) and the percentage occlusion of the T10 vertebra.

Spacers were designed based on T10 vertebral height, width, and length (Table 3). Although there were small statistically significant differences found in the measurements between P9 and P14 for vertebral height, once a suitable spacer was designed, the difference in spacer dimensions was <0.2 mm for these two age groups. For practical purposes a single “neonatal” spacer was developed based on the measurements for a P14 neonatal rat, and a single “adult” spacer was developed based on the average measurements for male and female rats (Figure 4).

### 3.2 Short‐term safety and survival studies

Surgery was performed on 36 (mixed‐sex) neonatal pups and 24 (12 M:12F) adult rats to assess safety and survival at 24 h, 3 days, and 7 days (*n* = 3–4 groups). Overall, there was a mortality rate of 6.6%. Two neonatal rats died due to anesthetics, and one was euthanized after rejection from the mother postsurgery. The procedures were revised to address both these issues. After spacer insertion, hindlimb deficits were observed in most animals, but there were no other neurological signs such as seizures or spasms observed over the maximum 7‐day period. All surviving rats were seen feeding and remained stable or gained weight postoperatively.

**FIGURE 5:**
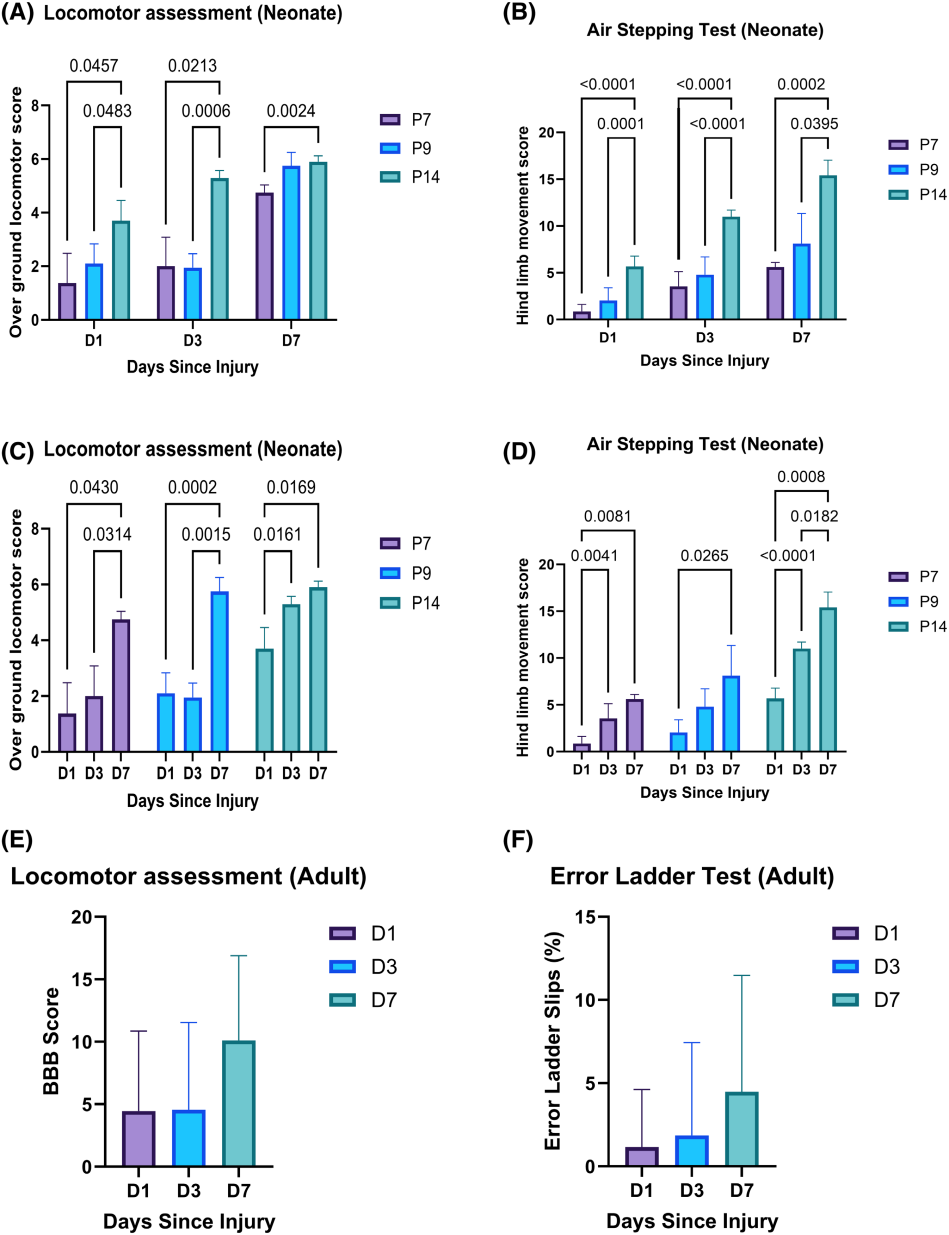
Behavioral results after 50% spinal cord compression in neonatal and adult Sprague–Dawley rats. Behavioral scores after T10 compression for 1, 3, and 7 days postinjury for neonatal rats at different postnatal ages (P7, P9, and P14) using (A and C) overground locomotor score for neonatal rats and (B and D) the air‐stepping task. Locomotive behavioral scores for adult rats are shown in (E) BBB (Basso, Beattie, and Bresnahan) hindlimb locomotor score and (F) the horizontal ladder stepping task. Analysis was conducted using two‐way ANOVA (analysis of variance) for neonates and one‐way ANOVA for adult rats, shown with mean ± standard deviation. Significant differences below *p* < 0.05 using Bonferroni post hoc tests are shown numerically.

For neonatal rats there was an increase in locomotor score over time and between different ages (two‐way ANOVA, *p*‐value as shown in Figure 5A–D). There were differences between P14 rats and P7 and P9 at both 1 and 3 days postinjury (Bonferroni post hoc tests, *p‐*values shown in Figure 5A). In the AS task, a similar pattern was observed with increases in locomotion over time and between different ages (two‐way ANOVA, *p*‐values shown in Figure 5B). There were significant differences between P7, P9, and P14 rats postinjury at 1, 3, and 7 days postinjury (Bonferroni post hoc tests, *p*‐values shown in Figure 5B). There were significant differences between days 1, 3, and 7 in the overground locomotor scores for all injury ages (Bonferroni post hoc tests, *p*‐values shown in Figure 5C), as well as a significant difference between days 1, 3, and 7 in the AS test for all injury ages (Bonferroni post hoc tests, *p*‐values shown in Figure 5D) as observed in Figure 5C,D. For adult rats (Figure 5E,F) there was no statistical difference in locomotor function at these early time points as scored in open‐field (BBB) and horizontal ladder tasks (% errors).

An examination of the spinal cord after euthanasia and dissection indicated the spacer was found to be in situ under the T10 vertebra in all animals. When the spacer was removed, a small distortion could be observed on the surface of the spinal cord.

### 3.3 Histology

**FIGURE 6:**
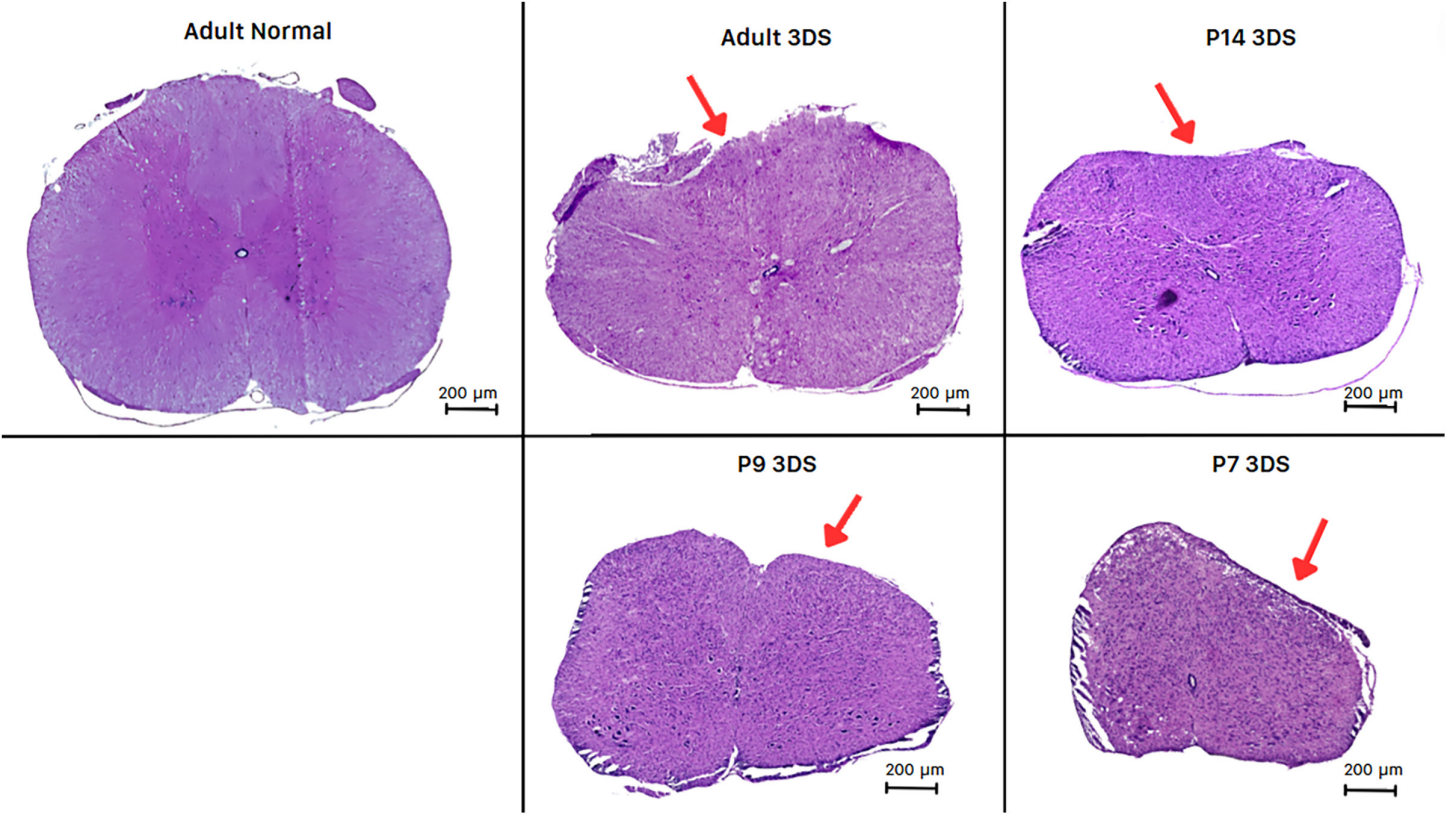
Impact of 50% compression using a solid spacer on the T10 spinal cord. Adult and postnatal days P7, P9, and P14 3 days after 50% compression spinal cord injury are shown with an H&E (hematoxylin and eosin) stain. A normal adult rat spinal cord section is shown for comparison. Red arrows indicate a compressed segment of injured spinal cords.

H&E sections confirmed deformation of the T10 spinal cord due to the spacer in each age group (Figure 6). When compared to normal sections, the dorsal part of the cord was depressed in animals that experienced spacer compression, as well as shifts and changes in the morphology of the central canal.

**FIGURE 7:**
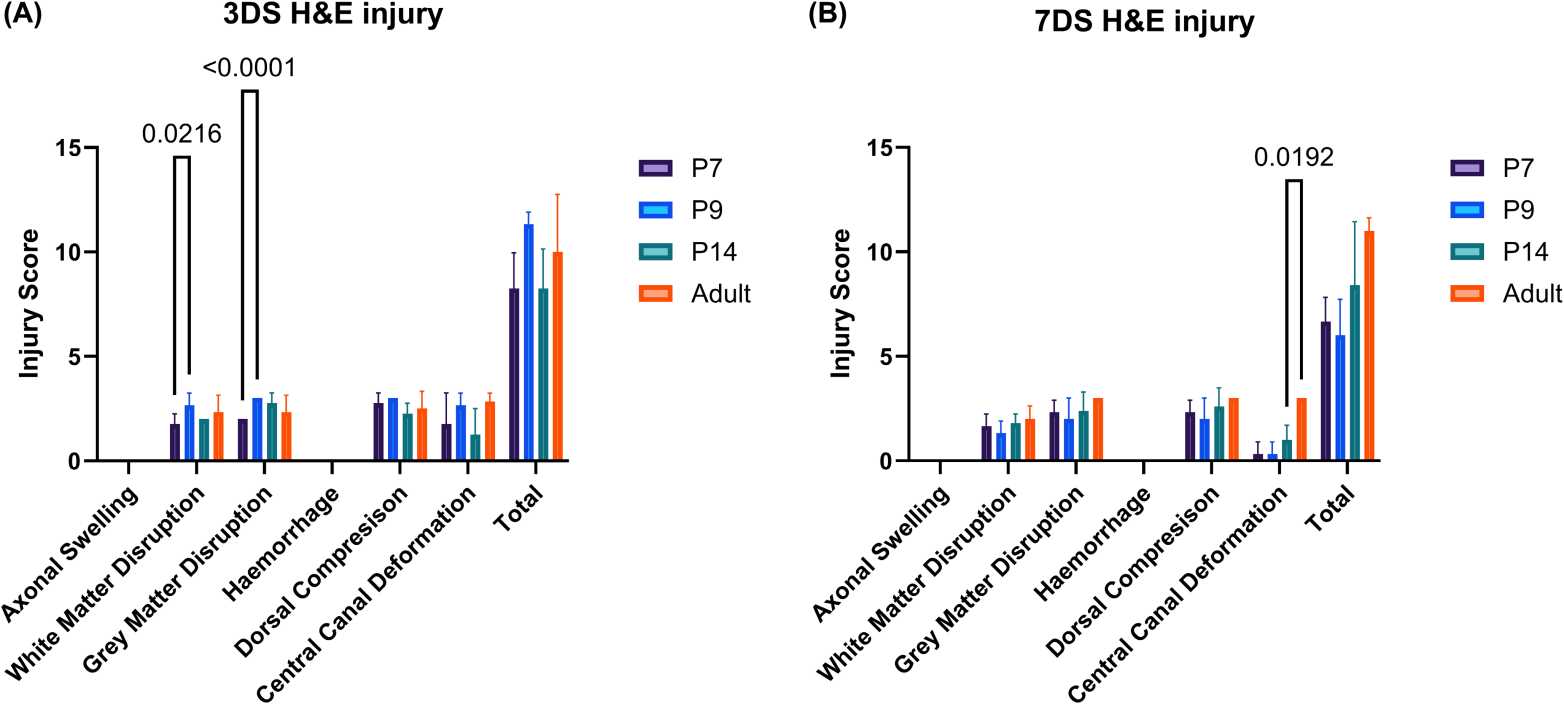
Analysis of injury severity at 3 days (A) and 7 days (B) postinjury using a 50% spinal cord compression injury in mixed‐sex postnatal day P7, P9, and P14, and 9‐week (adult) SD (Sprague–Dawley) rats. Injury severity was scored on a scale of 0–3, where 3 is the most severe level of injury. Disruption in six key areas was assessed to determine the severity of the injury. Analysis was performed using one‐way ANOVA (analysis of variance) and is shown with mean ± standard deviation. Significant differences below *p* < 0.05 using Bonferroni post hoc tests are shown numerically.

Additionally, there were disruptions to the white and gray matter, as presented in Table 1 (Figure 7). Significantly greater gray and white mater disruption occurred at P9 compared to P7 after 3 days of injury (Figure 7A). Central canal deformation was also significantly greater after 7 days of injury in adults compared to P14 (Figure 7B). No hemorrhaging was observed in any specimen 3 or 7 days postinjury, and no axonal swelling was observed at any time point or age. Analysis was performed to see if there was any correlation between the symmetry of injury, and the locomotor score is shown in Figure 5; 75% of these animals exhibited symmetry in their histology scores (left–right scores were within 1 point of each other). The maximum difference in scores between the left and right spinal cord was 3 points (5.6% of rats). There was no correlation between behavioral scores and the degree of asymmetry *R* ^2^ = 0.01 for any age, or obvious age differences between the groups. Adult rats exhibited the highest degree of asymmetry with 33% of 3‐day survival adults, and 83% of 7‐day survival adults with a degree of asymmetry >1 point difference between each hemisphere. Moreover, two‐way ANOVA with Bonferroni post hoc tests showed that there was no significant difference (*p* > 0.05) between 3‐ and 7‐day histology H&E injury scores for any measure.

## 4 DISCUSSION

The first aim of this project was to establish the dimensions needed for the design of an age‐ and sex‐specific spacer that would compress the SD neonatal and adult rat spinal cord by 50%. The second aim was to confirm that the insertion of this spacer did not have any acute off‐target effects, specifically that the spacer stayed in situ under the T10 vertebra and that there were no unexpected neurological events. The body weight of male and female SD rats is similar until ~28 days,^31^ and there is no indication that there would be any sex differences in vertebral column measurements for the neonatal rats. This was confirmed in our cohort, indicating that a single‐sized spacer is suitable for use in either sex up to 14 days. For adult SD rats, body weight starts to increase for males from ~4 weeks of age, and male rats are significantly larger and heavier than female rats by 9 weeks of age. However, at this age there were no statistically significant differences in the height or width of the T10 vertebral column in our study. It is not clear if the small increase in spinal cord area for adult male rats compared to adult female rats is biologically relevant as the percentage of spinal cord to canal was the same for all ages and sexes. As the dimensions of the spacer were calculated from height, width, and length of the T10 vertebral column and canal, it was determined that a single‐sized spacer is suitable for use in either sex in SD rats aged 9 weeks.

There was a significant increase in the size of the vertebrae between neonatal rats and adult rats, and this was expected, given the increase in size and weight of the animals over this time. There was also some variability in vertebral width and height between the P9 and P14 rats, which was statistically significant. However, taking these differences into account in the design and printing of the 3D spacers resulted in minimal differences in the actual size of the spacer and in fact could not be resolved by the printer itself.

In this type of model, it is important that the solid spacer does not move from the location under the T10 vertebra and does not cause acute injury to the spinal cord due to the surgical insertion itself. It should also not lead to any secondary downstream effects that are related to acute‐type injuries, which lead to further morbidity.^1^, ^7^, ^32^ First, the surgical insertion of the solid 3D printed spacer is an easy and gentle process. Once a laminectomy has been performed to remove the spinous process and lamina of the T11 vertebra, the spacer can be slid into position epidurally under the T10 vertebra with minimal pressure. All spinal cords were checked carefully for positioning during dissection, and the spacer was in situ for all. A small midline depression could be visualized on the surface of the spinal cord itself when the spacer was removed. There was no evidence of laceration, severe deformation, or bleeding on the surface of the spinal cord, and this was confirmed histologically.

This short‐term study was conducted to investigate the feasibility and safety of a solid spacer compression model in neonates and adult SD rats and therefore were not compared to an uninjured control group. Behavioral tests were included to assess the extent of hindlimb function in the short term. For the adult rats BBB scores on days 1, 3, and 7 are comparable to a 12.5‐mm weight decrease contusion injury, as described by Basso^29^ and commonly used in traumatic SCI research. Infant rat locomotor scores are more difficult to interpret as these animals do not exhibit mature locomotion at these ages, but the hindlimb function observed in this study is consistent with that reported for P7 SD rats in a previous mild contusion SCI study.^23^ The advantage of using a solid 3D printed spacer is that it is inexpensive and easy to produce, is readily adaptable to rats of different ages and species, and can be modified to produce graded injuries by increasing the percentage of spinal cord compression. We have shown that measurements of vertebral columns can be made on either formalin‐fixed dissected vertebral columns or intact vertebrae using MRI. The surgical procedures are also relatively simple, safe, and reproducible, even in very young animals, and result in measurable histological damage and behavioral function.

The disadvantage of using a solid 3D printed spacer for very young animals is the continuing development and growth of the spinal column, which will alter the eventual degree of spinal cord compression over time. This can be countered by increasing the initial spacer height to compensate for the increased height of the spinal canal with age. There is, however, no well‐characterized alternative chronic compression model available for very young animals. The majority of spinal cord compression models are conducted on adult rats, involve acute injures, or are part of combination trauma models.^15^ The underdevelopment of the vertebral column and the small size of neonatal animals mean that many techniques used in older animals are not suitable for infant animals.

Pediatric SCI is not well researched in part because there is no suitable model for very young animals. Children and infants tend to have more NTSCIs compared to adults,^11^, ^14^, ^33^ but this is not reflected in the current animal models of SCI. The lack of studies and data concerning pediatric SCI has been recognized,^6^, ^9^ as have many of the challenges involved in this type of research. It is expected that this solid 3D printed spacer model can be used to investigate the pathophysiology, temporal patterns, and eventual treatments for pediatric SCI.

## AUTHOR CONTRIBUTIONS

**Reggie Ridlen:** Data curation; formal analysis; investigation; methodology; writing – original draft. **Victoria Marsters:** Investigation; methodology. **Elizabeth Clarke:** Resources; software. **Kristine McGrath:** Resources; writing – review and editing. **Catherine A. Gorrie:** Conceptualization; funding acquisition; investigation; methodology; project administration; resources; supervision; validation; writing – review and editing.

## FUNDING INFORMATION

Reggie Ridlen has been supported by an Australian Government Research Training Program (RTP) scholarship, and funded by the University of Technology, Sydney.

## CONFLICT OF INTEREST STATEMENT

The authors declare that there are no conflicts of interest.

## ETHICS STATEMENT

These experiments had ethics approval from the University of Technology, Sydney animal care and ethics committee under ethics code ETH21‐6694.
